# Validation of a simplified small-scale DNA extraction protocol from wine by quantitative real-time PCR

**DOI:** 10.1007/s13205-024-03992-x

**Published:** 2024-05-02

**Authors:** Monica Scali, Giacomo Spinsanti, Rita Vignani

**Affiliations:** 1 Department of Life Sciences, University of Siena, 53100 Siena, Italy; 2 Department of Biotechnology, Chemistry and Pharmacy, University of Siena, 53100 Siena, Italy

**Keywords:** Wine DNA, *Vitis vinifera*, Real-time PCR, Molecular authentication, myIC

## Abstract

In the present study, we compared a simplified small-scale purification protocol to obtain DNA admixtures out of wine, with our large-scale published method. The extraction methods must provide DNA free of PCR inhibitors, that can interfere with DNA amplification. To evaluate the efficiency of grapevine’s nuclear DNA extraction from wine, the new protocol was also compared in terms of purity and yield to the DNA obtained out of grapevine’s (*Vitis vinifera*) leaf tissue, using a commercial kit. Two single-copy nuclear genes, nine-cis-epoxy carotenoid dioxygenase 2 (NCED2), and prefoldin subunit 5-like (PS5) were amplified in DNA extracted from wine and grapevine by real-time TaqMan PCR to determine the presence of inhibitors in relation to the diversity of starting biological matrix. This study showed that the small-scale, simpler method for extracting DNA from wine produced effective results in terms of inhibitor presence and purity. Furthermore, even though the initial biological matrix was more complicated, the grapevine nuclear DNA that was removed from wine was qualitatively equivalent to the DNA that was isolated from the leaves.

**Supplementary Information:**

The online version contains supplementary material available at 10.1007/s13205-024-03992-x.

## Introduction

In the last few years, the quality and safety of food products have become essential requirements to be guaranteed to consumers in all fields of agricultural production. The need to develop molecular analysis to confirm the authenticity of products and to prevent fraudulent actions has strongly increased after globalization (Scarano and Rao 2014). A plethora of analytical techniques, characterized by a high level of specificity, can be applied to raw materials or semi-processed products (Momcilovic and Rasooly 2000; Campos et al. 2023). While protein or proteomic-based approaches (immunological or electrophoretic assays) and metabolite screening (HPLC, or NMR profiling) are limited by uncontrolled effects due to environmental conditions and industrial procedures (Woolfe and Primrose 2004; Martinez and Friis 2004), the DNA-based methodologies seem to be more reliable and objective biotechnological tools to prove food matrixes’ composition and to prevent food and beverages adulteration. Despite important food-processing conditions, like mechanical, thermal, chemical, and enzymatic treatments, which may lead to the degradation of the DNA, it can be still suitable to be used in PCR-based authentications (Hird et al. 2006). Although PCR is widespread in the field of food testing, it raises problems and difficulties especially when heterogeneous matrices or processed food are analyzed (Bottero and Dalmasso 2011). The type of sample used for DNA extraction can influence amplification reaction as DNA quantity and quality often vary accordingly (Chaudhry et al. 1999). The aim of a nucleic acid extraction method is to isolate DNA of suitable integrity, purity and of sufficient quantity for PCR amplification and other possible downstream research applications (Terry et al. 2002). DNA extracted from food products tends to be low in quantity and to be highly degraded in relation to the extent to which the food has been processed. Generally speaking, exposure to heat results in the fragmentation of high-molecular-weight DNA, and physical and chemical treatments cause random breaks in DNA strands, reducing fragment size (Di Bernardo et al. 2008). For all these reasons, the scientific community is constantly looking for DNA extraction protocols simple, quick, and efficient, taking into consideration safety, costs, and DNA quality (Abriouel et al. 2006; Cankar et al. 2006).

The wine sector is a major economic activity, and the selection of grape varieties is of primary concern for wine quality. Wine DNA authentication is a technological process that addresses at verifying the biological nature of the grapevines used (Galstyan et al. 2021; Lazareva et al. 2020; Oganesyants et al. 2018). Various methods are available for the extraction of DNA from musts and commercial wines, as well as molecular approaches to the genetic identification of grape varieties which wines are made from Vignani et al. (2019), Pereira et al. (2018), Scali et al. (2014), Bigliazzi et al. (2012), Recupero et al. (2013) and Barrias et al. (2019). To analyze the must or wine-extracted DNA for traceability purposes, several studies have been carried out using molecular markers: the majority are based on microsatellites and SNPs (Zambianchi et al. 2021). The extraction of DNA from wine is the starting point for downstream molecular biology analysis, and the success of a PCR reaction is dependent on the quality of genomic DNA, highlighting the importance of quick and efficient DNA extraction methods (Brent et al. 2002). Due to complicating factors such as DNA decomposition during fermentation, interference of DNA extraction by polysaccharides and polypeptides in the wine, and the coexistence of pigment substances such as polyphenols, which inhibit DNA polymerase for PCR, molecular characterization of grape cultivars by PCR using wine as a sample can be difficult (Gao et al. 2015; Nakamura et al. 2007; Siret et al. 2000; Miller et al. 1999).

The greatest difficulty in establishing a molecular traceability protocol for wine is consolidating efficient techniques for extracting DNA fractions. The purification of DNA out of wine remains the crucial bottleneck of the entire molecular traceability procedure. The amount of DNA in wine can be very low, and the analysis of genotyping reconstruction is complicated by the nature of wine DNA that derives from multiple biological contributors, and it is generally chemically and physically compromised due to wine-making procedures such as fermentation, aging, clarification, and filtration (Gao et al. 2015). They can affect downstream applications, leading sometimes to underestimated DNA concentrations, and false-negative results (Funes-Huacca et al. 2011; King et al. 2009; Kontanis and Reed 2006).

Different approaches can be used to improve the downstream applications performance of purified DNAs, such as removing inhibitors during DNA extraction and purification, and reducing the effects of inhibitors by later manipulation of template DNA or PCR reagents (Alaeddini 2012; Kemp et al. 2006). These methods, however, tend to determine a significant loss of DNA quantity.

Therefore, it is preferable to choose techniques that decrease time and cost, eliminating as many inhibitors as possible, while maintaining DNA yield and purity (Monroe et al. 2013). The choice of the DNA extraction protocol is a crucial point in wine molecular analysis and critically determines the performance of the downstream PCR applications, including DNA quantification and subsequent wine DNA fingerprinting (WDF) via SSR amplification for the varietal assessment (Vignani et al. 2019). Historically, cultivar identification of wines using DNA biomarker technology was problematic due to the very low level of DNA fragments in processed wines. However, recent advances in the extraction protocol of DNA fragments and the utility of PCR technique have allowed researchers to overcome sensitivity issues in DNA analysis in wines (Vignani et al. 2019). In this context, the amount and the purity of *Vitis vinifera* genomic DNA that can be extracted from wine samples is an essential condition for varietal composition validation of different wines with increasing varietal complexity.

In the present study, we compared, based on the yield and purity of the final product, a simplified small-scale DNA extraction protocol from wine, with our published method of wine DNA extraction (TECP method, Bigliazzi et al. 2012; Scali et al. 2014), and a genomic DNA extraction protocol for plant tissues (DNasy Plant Mini Kit—Qiagen). Seven single-copy nuclear genes were tested on different extractions of genomic *Vitis vinifera* DNA, and one was selected based on the yield in RT-PCR reactions: prefoldin subunit 5-like (PS5). PS5 gene and nine-cis-epoxy carotenoid dioxygenase 2 (NCED2) gene, already described as a single-copy target in the *Vitis vinifera* genome (Savazzini and Martinelli 2006), were amplified in DNA extracted from wine and grapevine in a real-time multiplex PCR. An exogenous standard DNA (myIC) (González-Escalona et al. 2009) served as an internal quantitative and qualitative control. Both myIC and *Vitis vinifera* genomic DNA from plant and wine were compared by absolute RT-PCR, demonstrating that the simplified small-scale DNA extraction procedure from wine generates efficient results in terms of purity and inhibitor presence.

## Materials and methods

### Grapevines and wines samples

Plants and wines analyzed in this study are listed in Table 1: seven grapevines including three genetic variants of Merlot and Sangiovese, one Cabernet Sauvignon, and two monovarietal Sangiovese wines. The Merlot and Cabernet Sauvignon grapevines were obtained by the National Grapevine Repository collection at Conegliano Veneto (CREA-VIC), while the Sangiovese samples (grapevines and monovarietal wines 0372 and 0373) were kindly provided by local winemakers (Caprili and Col D'Orcia, respectively, Montalcino, Siena, Italy).

**Table 1:** List of grapevines and wines with abbreviations

Samples	Abbreviation	Wine	Plant
Merlot	Merlot_1		X
Merlot	Merlot_2		X
Merlot	Merlot_3		X
Cabernet Sauvignon	CB		X
Sangiovese	SG		X
Sangiovese	0372	X	X
Sangiovese	0373	X	X

### DNA purification from grapevine leaves

Plant DNA was extracted from grapevine leaves using the commercial DNeasy Plant Mini Kit (QIAGEN, Germany), following manufacturer instructions. Homogenization of plant tissues was obtained by grinding 100 mg of fresh young leaves. Final DNA samples were eluted in 100 μL of Buffer AE (10 mM Tris–HCl; 0.5 mM EDTA; pH 8.0).

### DNA purification from wine by TECP method

The TECP method (TRIS–EDTA-CTAB-PVPP) used for DNA extraction from wine was described by Bigliazzi et al. (2012), and subsequently updated by Scali et al. (2014). The wine sample (approximately 300–400 mL) was precipitated by adding 1 volume of 0.3 M sodium acetate (3 M, pH 5.2) and 1 volume of isopropanol, and it was kept at − 80 °C for at least 3 days.

### Simplified small-scale DNA extraction protocol from wine

**Fig. 1:**
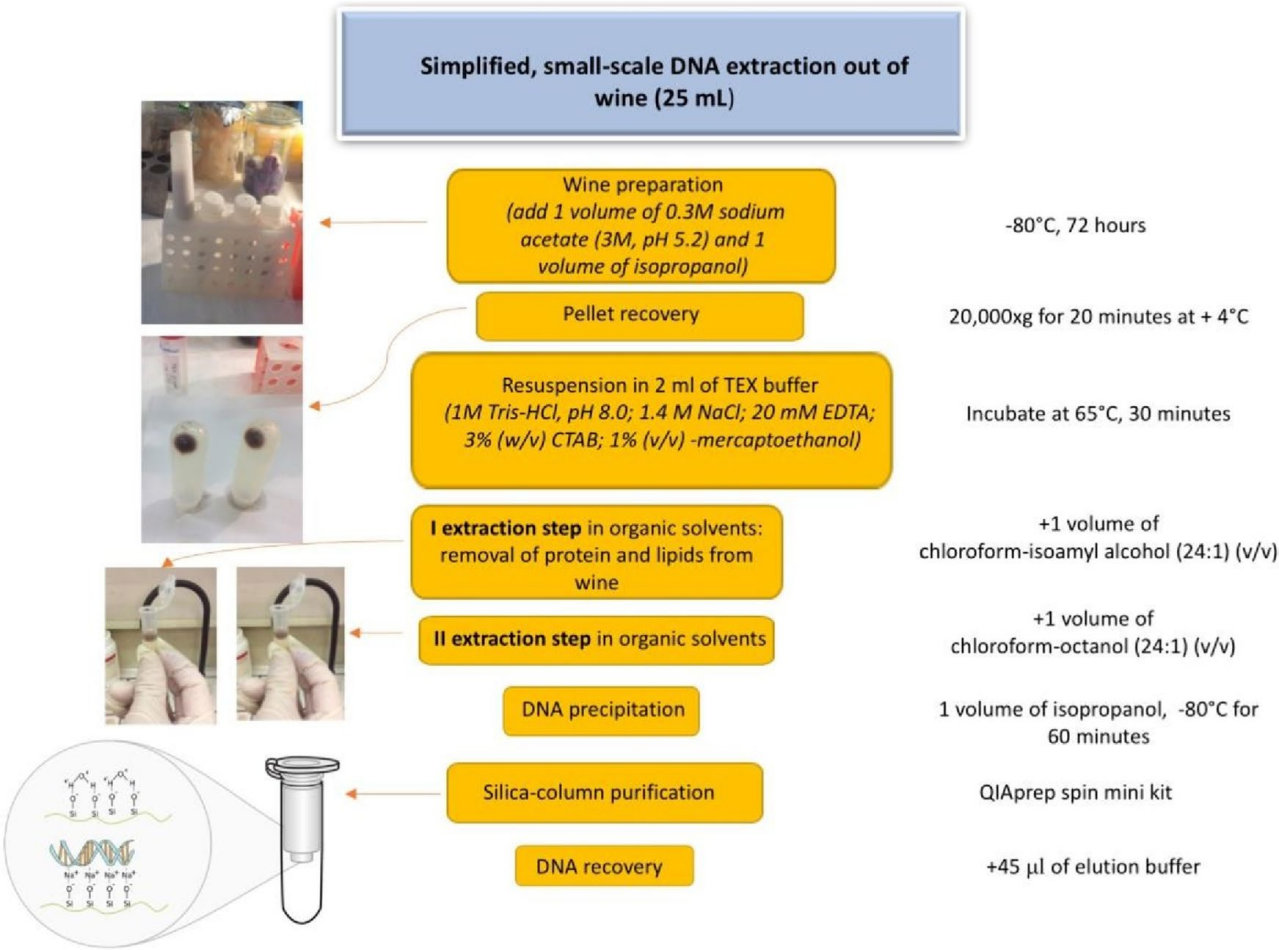
Workflow of a simplified small-scale DNA extraction protocol out of wine. A visual summary of the protocol is depicted and the main steps include: incubation at − 80 °C followed by two extractions in organic solvents, and a final purification using silica columns

This protocol is a simplified version of TECP method. Before processing, wine sample (approximately 20–25 mL) was precipitated by adding 1 volume of 0.3 M sodium acetate (3 M, pH 5.2) and 1 volume of isopropanol, and it was kept at − 80 °C for at least 3 days. After precipitation,

samples were centrifuged at 20,000 *g* for 20 min at 4 °C. The pellets were resuspended in 2 mL of TEX buffer (1 M Tris–HCl, pH 8.0; 1.4 M NaCl; 20 mM EDTA; 3% (w/v) CTAB; 1% (v/v) β-mercaptoethanol), and were incubated for 30/40 min at 65 °C. Tubes were cooled at room temperature and the first organic solvent extraction was performed by adding 1 volume of chloroform–isoamyl alcohol (24:1) (v/v). Samples were vortexed for 1 min and centrifuged at 5.000 *g* for 10 min at 4 °C. Supernatants were recovered and a second organic solvent extraction was performed by adding 1 volume of chloroform–octanol (24:1) (v/v). Samples were vortexed for 1 min and centrifuged at 5.000 *g* for 10 min, at 4 °C. Then 1 volume of isopropanol was added to precipitate samples at − 80 °C for 60 min. After precipitation, samples were centrifuged at 14.000 *g* for 30 min, at 4° C. The pellets were resuspended in 250 μL of Buffer P1 (50 mM Tris- HCl pH 8.0; 10 mM EDTA; 100 μg/mL RNaseA) and 4 of proteinase K (20 mg/mL). Samples were incubated for 10 min at 50 °C. The extraction was completed following the QIAprep Spin Miniprep Kit (QIAGEN- Germany). DNA samples were eluted in 45 μL of elution buffer (10 mM Tris–HCl; 0.5 mM EDTA; pH 8.0) and stored as such at − 20 °C (Fig. 1).

### DNA quantification by spectrophotometer

DNAs obtained from Merlot grapevines and Sangiovese wines using the two different extraction procedures were quantified using the NanoDrop™ 1000 (Thermo Fisher Scientific) and the Infinite® 200 PRO NanoQuant (Tecan).

### Selection of single-copy nuclear genes in *Vitis vinifera*

A set of single-copy nuclear genes common to genera Arabidopsis, Populus, Vitis, and Oryza were selected from the literature (Duarte et al. 2010) (Table 2). The single-copy nuclear genes were tested in real-time SYBR Green PCR assays and compared to the NCED2 gene, already described as a single-copy target in the *Vitis vinifera* genome (Savazzini and Martinelli 2006).

**Table 2:** Single-copy nuclear genes shared between *Arabidopsis thaliana* and *Vitis vinifera*

*Arabidopsis* Locus ID	*Vitis vinifera* Locus ID	Annotation *Vitis vinifera*
At2 g21870	LOC100254399	ATP Synthase subunit 24 kDa, mitochondrial
		(*ATPSynth24*)
At4 g33250	LOC100259495	Eukaryotic translation initiation factor 3 subunit K
		(*ETIF3*)
At4 g30010	LOC100264401	Ubiquitin-fold modifier 1 (*UBFM1*)
At4 g31720	LOC100248501	Transcription initiation factor TFIID subunit 12b (*TF2D*)
At5 g47570	LOC100246750	NADH dehydrogenase [ubiquinone] iron-sulfur protein 1
		(*NadhDISP1*)
At5 g23290	LOC100243012	Prefoldin subunit 5-like (*PS5*)
At1 g27530	LOC100264226	Ubiquitin-fold-modifier-conjugating enzyme 1
		(*UBFMCE1*)

The Locus ID and the abbreviations are reported

### Primers and probes

The software Beacon Designer (Premier Biosoft) was used to design the primers and the probes sequences (Table 3). All primers (desalted, 0.02 μM) and probes (HPLC purified, 0.04 μM) were from Bio-Fab Research. The specificity of each oligo was confirmed by matching them in the Genbank database using BLAST (www.ncbi.nlm.nih.gov/BLAST).

**Table 3:** List of primers and probes sequences (F: forward; R: reverse)

Primer/probe	Oligonucleotide sequence 5ʹ–3ʹ	Amplicon length (bp)	Length (bp)	Note
ATPSynth24	GCAGACTTGTTTTCAGAATCC		21	F
ATPSynth24	TCTCCTTCAATGTCAACAGATATG		24	R
ETIF3	TCAGGAATGTTAAGAGAC		18	F
ETIF3	ATCAGCACAATATACCAT		18	R
UBFM1	TGAAGAGATTGGACTACATT		20	F
UBFM1	GATGCTCAGGAGAGTATG		18	R
TF2D	CATGTCAAGGATCAATTC		18	F
TF2D	GAAGTTCATCTGGCTATA		18	R
NadhDISP1	ATGGGGTTAGGGTTGATA		18	F
NadhDISP1	TGATGGTTCTGGTTATAGG		19	R
UBFMCE1	AGTACAAGGCATTGATAG		18	F
UBFMCE1	TTGAGGAGATTATGAACAT		19	R
PS5	GACAGATCTCGAGGTCAA		18	F
PS5	GCACCAACATCTTCTTCC	130	18	R
PS5	[HEX]CCTCAACAACATCCGCACCG[BHQ1]		20	Probe
NCED2	GCCTCCTCCTCTTCTATG	100	18	F
NCED2	AGCCTCTGATTGAAGTACA		19	R
NCED2	[Cyc5]CAAGCCAGCGTTAGCCACTC[BHQ2]		20	Probe
myIC	CTAACCTTCGTGATGAGCAATCG		23	F
myIC	GATCAGCTACGTGAGGTCCTAC		22	R
myIC	[6Fam]AGCTAGTCGATGCACTCCAGTCCTCC		27	Probe
T[BHQ1]				

### SYBR Green real-time PCR

Total DNAs obtained from each Merlot, Sangiovese and Cabernet Sauvignon leaves were used to confirm single-copy nuclear genes using SYBR Green real-time PCR assays. The final reaction mixture contained 10 μL of SYBR^®^ Green master mix (dNTPs, MgCl_2_, and DNA polymerase) (Bio-Rad), 0.6 μL of each primer, 6.8 μL of H_2_O, and 1 μL of DNA. The final concentration of primers in the qRT-PCR mix was 300 nM. The reactions were performed in a CFX96 Real-Time detection system (Bio-Rad) in a volume of 20 μL in triplicate, under the following conditions: 95 °C for 3 min, 95 °C for 10 s, 55 °C for 30 s, repeat step 2 and 3 for 39 cycles, 95 °C for 10 s, 65 °C to 95 °C incrementing 0.5 °C for 5 s. The last step corresponds to the melting curve analysis.

### TaqMan real-time PCR

DNAs isolated from the Merlot leaves and Sangiovese wines were also used to perform co-amplifications with an exogenous control DNA myIC in TaqMan real-time PCR assays. The final reaction mixture contained 10 μL of SsoFastTM Probes Supermix (Bio-Rad), 0.6 μL of each primer, 0.4 μL of each probe, 4.8 μL of H_2_O, and 1 μL of DNA. The duplex TaqMan Real Time-PCR reactions contained 2 μL of template DNA (1 μL of sample DNA and 1 μL of myIC DNA as standard). The final concentrations of primers and probes in the qRT-PCR mix were, respectively, 300 nM and 200 nM. Reactions in a final volume of 20 μL were performed in triplicate using a CFX96 Real-Time detection system (Bio-Rad) under the following conditions: 95 °C for 3 min, 95 °C for 10 s, 55 °C for 1 min, repeat steps 2 and 3 for 39 cycles.

### myIC calibration curve

The myIC primers and probe sequences for qPCR analysis were from the literature (González-Escalona et al. 2009) (Table 3). The myIC sequence, a synthetic construct that does not match any currently available sequence in the Genbank database (http://www.ncbi.nlm.nih.gov/), is deposited under the accession number FJ357008.

The myIC calibration curve was determined on a Bio-Rad CFX96 system using six serial dilutions (1:10) of myIC DNA (Bio-Fab Research) at an initial concentration of 0.01 pM (1 × 10^–8^ μM) (Figs. 4 and 5).

### Absolute quantification of *Vitis vinifera* genomic DNA

A myIC dilution to 1 × 10^–10^ µM was used as internal standard control and it was co-amplified in two duplex TaqMan assays: the former with NCED2 and the latter with PS5 genes. The baseline thresholds, based on signal-to-noise ratios over a set number of cycles, of myIC, NCED2, and PS5 have been set to a determined shared value. The quality and quantity of DNA were, therefore, evaluated by normalizing the Ct values obtained by a comparison between the NCED2 and myIC amplifications applying the formula of 2^Δ^Ct.

The internal standard myIC used in duplex TaqMan real-time PCR corresponded to a concentration of 6.2 × 10^–9^ ng/µL. The copy number of myIC was, therefore, calculated using the copy number calculation factor for real-time PCR (http://scienceprimer.com/copy-number-calculator-for-realtime- pcr).

A *Vitis vinifera* haploid genome is 487Mbp (Jaillon et al. 2007), making 951 copies of diploid genomic DNA in 1 ng (http://cels.uri.edu/gsc/cndna.html).

$${951}\;copies:{1}000 {\text{pg}} = {1}:x\;pg.$$

Thus, the weight of one double-strand *Vitis vinifera* genome can be estimated as 1.05 pg.

### NCED2 relative normalized expression

The relative normalized expression of NCED2 was calculated using the Bio-Rad CFX96 Manager software, version 3.1, which is based on an optimized calculation of the 2^ΔΔCt^ method, assuming myIC as a reference gene.

## Results and discussion

### Evaluation of single-copy nuclear genes

**Fig. 2:**
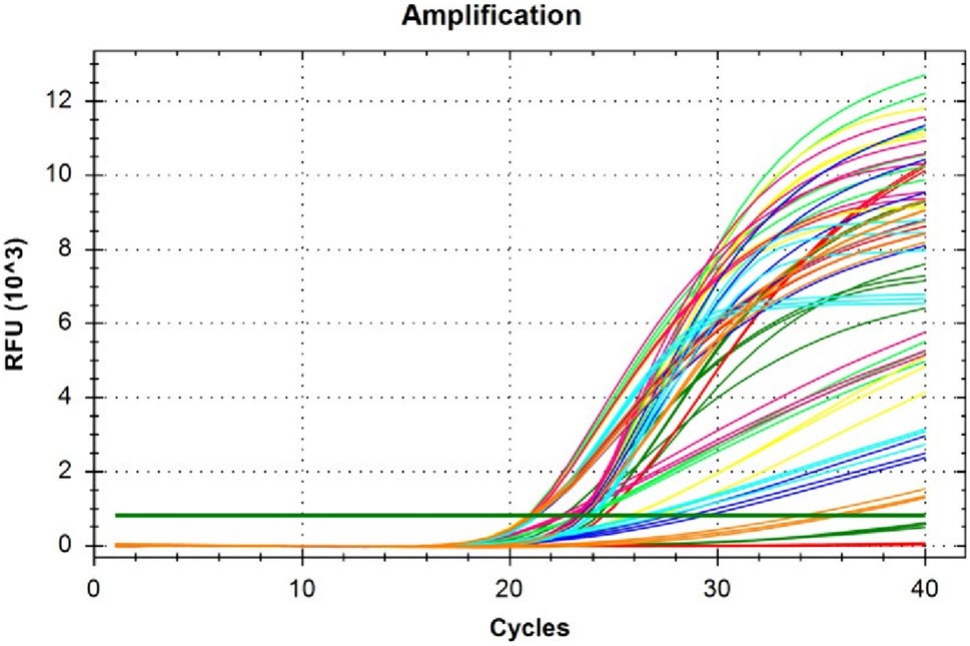
Seven genes were tested in real-timePCR, using SYBR Green as a fluorescent dye, on different DNA extractions from grapevine cultivars (Merlot, Sangiovese, and Cabernet Sauvignon) to verify their copy number. The genes analyzed were the following: UBFM1 (dark green), ETIF3 (red), PS5 (light green), TF2D (yellow), NadhDISP1 (blue), ATPSynth24 (light blue) and UBFMCE1 (orange). NCED2 (fuchsia) was used as a single-copy gene control

**Fig. 3:**
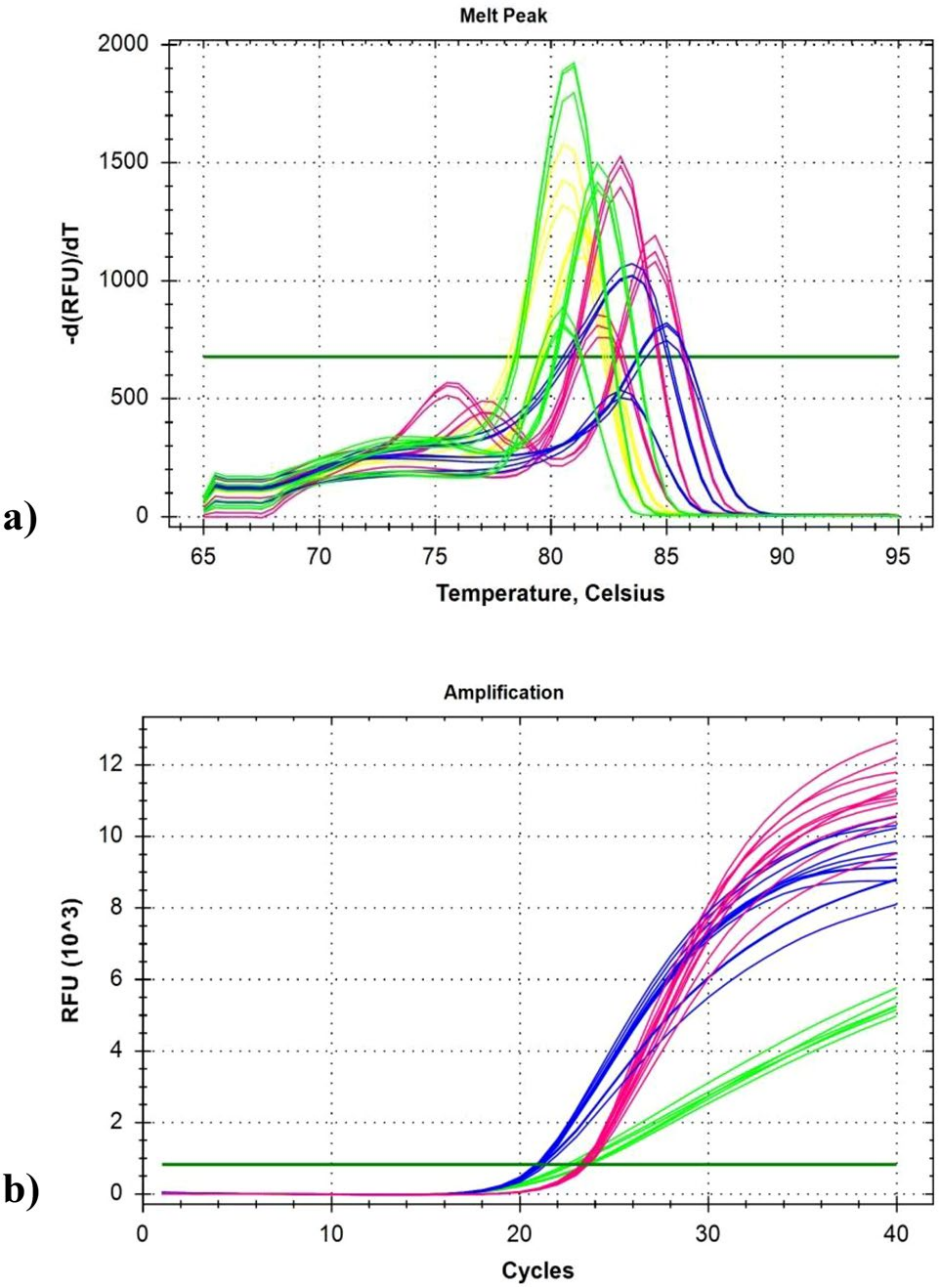
**a** Amplifications of the selected genes (PS5, TF2D, and NadhDISP1) in real-time PCR on three different DNA extractions from plant tissue: Merlot (light green), Sangiovese (fuchsia), and Cabernet Sauvignon (blue) producing highly comparable Ct values within each sample, perfectly matching those recorded for NCED2, confirming to be single-copy nuclear genes; **b** melting curves of the NCED2 (fuchsia), PS5 (light green), TF2D (blue), and NadhDISP1 (yellow) amplicons demonstrated the uniqueness of the PCR amplifications in all samples

A new simplified version of DNA extraction protocol from wine has been evaluated developing a quantitative real-time PCR method to determine the amount and purity of *Vitis vinifera* genomic DNA. Single-copy nuclear genes allow relating the starting concentration of DNA to the number of amplicons at the end of the PCR reaction. For this purpose, it was necessary to test single-copy number nuclear genes of *Vitis vinifera*, to be targeted in real-time PCR as quantitative markers of genome number in DNA admixtures purified from wine. Seven single-copy nuclear genes (Table 2) were tested in SYBR Green real-time PCR assays performed on grapevine cultivars (Merlot, Sangiovese, and Cabernet Sauvignon) in addition to NCED2, already described as a single-copy target in the *Vitis vinifera* genome (Fig. 2). Among the seven single-copy nuclear genes analyzed, TF2D, PS5, and NadhDISP1 (Fig. 3a) produced highly comparable Ct values within each sample, perfectly matching those recorded for NCED2, confirming to be single-copy nuclear genes (Table 4).

**Table 4:** Ct values of NCED2, PS5, TF2D and NadhDISP1 genes and ΔCt values calculated with respect to NCED2 Ct

Samples	NCED2 Ct	PS5 Ct	ΔCt	TF2D Ct	ΔCt	NadhDISP1 Ct	ΔCt
Merlot	20.5	20.57	0.07	20.5	–	20.53	0.03
Cabernet Sauvignon	21.02	20.9	0.12	20.88	0.14	21.22	0.2
Sangiovese	29.21	30.10	0.89	32.3	3.09	30.57	1.36

The melting curves (Fig. 3b) demonstrated the uniqueness of the PCR amplifications in all samples.

Based on their amplification efficiencies and Ct values, NCED2 and PS5 were selected for further quantitative analysis developing two duplex TaqMan real-time PCR assays consisting of simultaneous amplification of myIC plus NCED2 and myIC plus PS5. Among the two duplex TaqMan real-time PCR assays, the myIC/NCED2 was preferred over the myIC/PS5 due to its better technical performance, even though the myIC/PS5 showed to retain good technical efficiency properties.

### Spectrophotometer quantification of *Vitis vinifera* DNA extracted from Merlot grapevines and Sangiovese wines

The DNAs extracted out of Merlot grapevines and monovarietal Sangiovese wines using the two different protocols were quantified using two different spectrophotometric systems. The two systems produced dissimilar results among all samples (Table 5). Conversely, values of the absorbance ratio at 260/280 appeared to be highly comparable, hovering around the optimal value of 1.8 (indicating the absence of proteins, phenols, or other contaminants). Despite overall differences, it is possible to observe a constant ratio between grapevine DNAs: in both measurements, Merlot_1 appears to be five times more concentrated than Merlot_2 and Merlot_3 which share less dissimilar measures. The higher variability of quantifications of wine DNA samples may be linked to the use of CTAB during the extraction protocol, which could influence the absorbance ratios in the spectrophotometric readings. However, it has to be noted that the spectrophotometric DNA quantification refers to the total DNA in the sample: in the case of the plant leaf, the total DNA includes mitochondrial DNA, plastid DNA, and genomic DNA, while in the case of wine, it includes residual DNA of bacteria and yeasts, in addition to the *Vitis vinifera* total DNA.

**Table 5:** Comparison between two different spectrophotometric quantifications of DNA extracted from Merlot plants (Merlot_1, Merlot_2, and Merlot_3) and Sangiovese monovarietal wines (0372, 0373)

Samples	Origin	NanoDrop ng/μL	O.D. 260/280	NanoQuant ng/μL	O.D. 260/280
Merlot_1	Leaf	159.93	1.92	134	1.9
Merlot_2	Leaf	30.2	1.8	25.5	1.81
Merlot_3	Leaf	23.81	2.09	39.5	1.87
SP_0372	Wine	49.64	1.77	30.2	1.78
LB_0372	Wine	16.25	1.5	17.7	1.59
SP_0373	Wine	13.64	1.6	61.6	1.59
LB_0373	Wine	25.95	1.69	65.7	1.81

The prefix SP refers to DNA extracted using simplified small-scale protocol and LP to DNA extracted using TECP method, respectively

### Duplex TaqMan real-time PCR

**Fig. 4:**
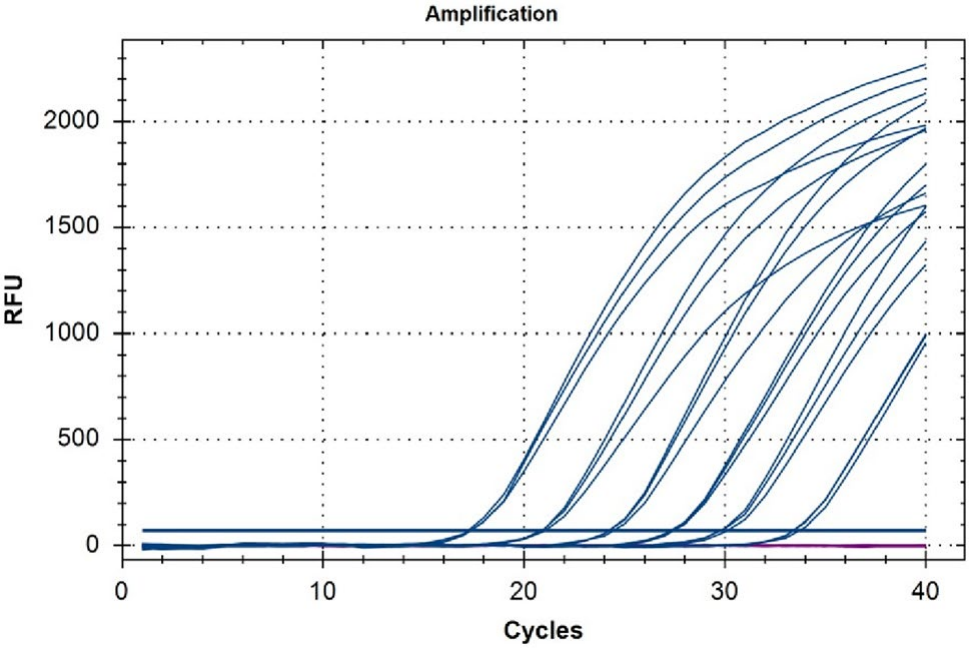
Six serial (1:10) dilutions of exogenous DNA myIC (blue) were tested in TaqMan real-time PCR in duplex with NCED2 primers and probe (violet). Among the serial dilutions, the 1 × 10^–10^ μM was selected as the internal quantification control in the duplex TaqMan real-time reactions with NCED2 and PS5

**Fig. 5:**
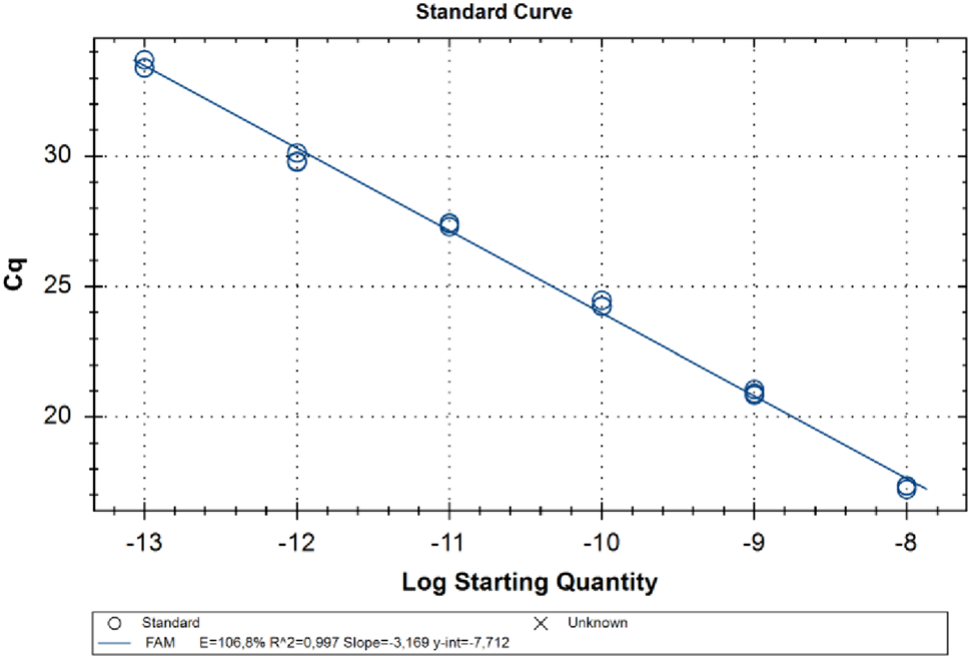
The calibration curve was obtained from serial dilutions of myIC. The amplification reaction shows optimal efficiency of 106.8% and reproducibility with a calculated *R*^2^ value of 0.997

The exogenous DNA myIC with its specific primers and TaqMan probe was used to design a calibration curve (Figs. 4 and 5) using six serial 1:10 dilutions starting from the concentration of 0.01 pM, that corresponds to 1 × 10^–8^ μM. The amplification reaction shows optimal efficiency of 106.8% and reproducibility with a calculated *R*^2^ value of 0.997 (Wacker and Godard 2005). Among the serial dilutions, the 1 × 10^–10^ μM was selected as the internal quantification control in the duplex TaqMan real-time reactions with NCED2 and PS5.

Two duplex TaqMan real-time PCR assays have been accurately developed for the simultaneous amplification of each one of the selected single-copy genes together with the internal control. The assays have been tested on the DNAs isolated from the different samples (Table 1) and the NCED2/myIC co-amplification reaction was preferred in terms of efficiency and reproducibility to PS5/myIC co-amplification (data not shown).

**Fig. 6:**
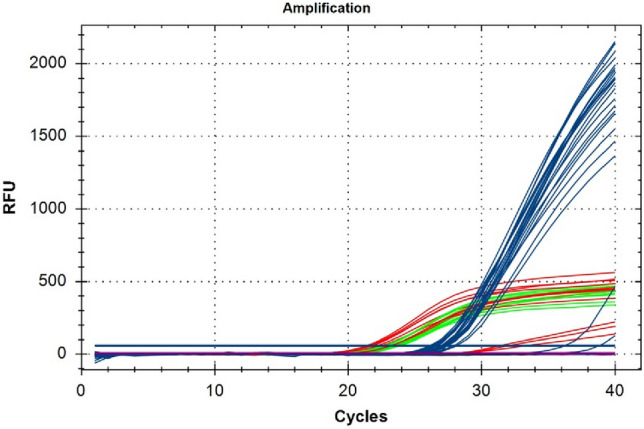
Duplex TaqMan real-time PCR with myIC (blue) and NCED2 (green for plants and red for wines). Samples are *Vitis vinifera* DNA extracted from Merlot plants (green) and DNA extracted from the monovarietal Sangiovese wines (red) using the two different protocols. Data show how both DNAs from Merlot plants and Sangiovese wines are comparable in terms of yield

One microliter of myIC at the concentration of 1 × 10^–10^ μM was added to the reaction mix together with one microliter of template DNA isolated from Merlot plants and Sangiovese wines. The co-amplification of myIC together with the selected single-copy gene NCED2 allowed us to determine the possible presence of inhibitors affecting the reaction and gave information about the amount of *Vitis vinifera* genomic DNA. The comparison between Ct values of myIC and NCED2 pointed out the supposed concentration and copy number of *Vitis vinifera* genomes. Data show how both DNAs from Merlot plants and Sangiovese wines are comparable in terms of yield except for sample LP_0372 (Fig. 6). The amplification of the myIC shows a tight range of Ct (between 26.13 and 28.13) indicating that the same concentration of inhibitors is contained in all samples. The worst performance of LP_0372 can, therefore, not be related to the presence of contaminants but could be due to problems related to the degradation of the sample.

### Absolute and relative quantification of extracted *Vitis vinifera* genomic DNA

Assuming that the amplification efficiencies of both myIC and NCED2 were similar, absolute quantification of *Vitis vinifera* genomic DNA extracted from plants and wines was made. For each sample, the Ct values of NCED2 were referred to those of myIC, co-amplified in the same reaction, and the exact quantity of genomic DNA was retrieved applying the 2^ΔCt^ formula considering the myIC Ct value corresponding to 29.64 pg (Table 6).

**Table 6:** Absolute quantification of *Vitis vinifera* DNA (pg/μL) from Merlot plants and Sangiovese wines

Samples	NCED vs myIC	pg/μL DNA of *Vitis vinifera*
Merlot_1	27.8 X	824
Merlot_2	10 X	296.4
Merlot_3	25.1 X	744
SP_0372	56.1 X	1662.8
LP_0372	− 21 X	1.41
SP_0373	16.8 X	497.97
LP_0373	11.71 X	347.1

**Fig. 7:**
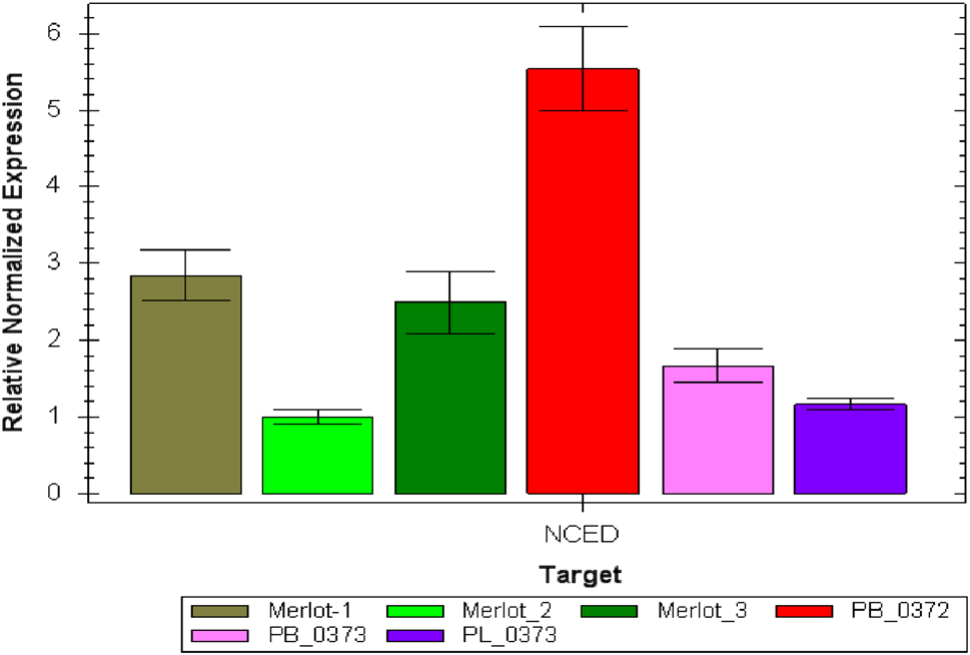
Relative normalized quantification of NCED2 gene in all samples excluding PL_0372 that produced DNA fractions below the analytical threshold limit of forensic practices. The reference gene is myIC and the control is Merlot_2 (light green). Data reported in the graph show that both protocols from wine: the simplified small-scale protocol and the TEPC method can isolate comparable yields of genomic DNA, capable of reliable performances in real-time PCR

A relative quantification was also made by applying the 2^ΔΔCt^ method using myIC as a reference gene and the Merlot_2, having the lowest Ct value after normalization, as the control (Fig. 7). It is clear from the graph that both protocols from wine: the simplified small-scale protocol and the TEPC method can isolate comparable yields of genomic DNA, capable of reliable performances in real-time PCR. The *Vitis vinifera* genomic DNA obtained from wines, in terms of quantity, was far above the theoretical limit of low DNA template quantity corresponding to 12.5 pg, as defined in forensic science (Bessekri et al. 2015; Vignani et al. 2019), and concerning downstream application potential, agrees with the literature (Pereira et al. 2017; Bigliazzi et al. 2012) proving the validity of the use of the DNA-based methods for wine varietal authentication.

## Conclusion

In the present study, we checked the efficiency of a simplified small-scale DNA extraction protocol from wine. The evaluation of the protocol was mainly based on its capacity to purify suitable *Vitis vinifera* DNA for wine DNA Fingerprinting (WDF) (Vignani et al. 2019) in terms of yield and efficiency.

According to the real-time PCR performances, all seven nuclear genes tested in our study were confirmed to be present as single copy of the *Vitis vinifera* genome. Due to its amplification efficiency and reproducibility, NCED2 has been selected to develop a new quantitative method for the quantification of *Vitis vinifera* genomic DNA, both in wines and grapevine varieties. This method proved that the simplified small-scale DNA isolation protocol from wine is comparable to the published TEPC one (Bigliazzi et al. 2012; Scali et al. 2014), for yield and purity. It has also been demonstrated that comparable quantities of DNA can be obtained starting both from wine and leaf tissue.

This type of analysis represents an innovative selective method to quantify the genomic DNA of *Vitis vinifera* in a sample, independently from the presence of undesired DNA derived from plastids or yeasts. The amount of *Vitis vinifera* DNA obtained by the wine DNA extraction protocols resulted in being abundantly above the threshold minimal quantity of 12.5 pg of template DNA, below which, according to forensics medicine, stochastic effects occur during the PCR amplification process (Bessekri et al. 2015; Vignani et al. 2019).

Therefore, the simplified small-scale protocol for wine DNA extraction, being less laborious and expensive, represents a valid alternative to obtain high-quality DNA admixtures suitable for molecular authentication methods.

### Supplementary Information

Below is the link to the electronic supplementary material.

Supplementary file1 (TIF 131 KB)

Supplementary file2 (TIF 153 KB)

## Data Availability

The authors confirm that all data generated or analyzed during this study are available within the article. The authors declare that the research does not involve human participants or animals.
