# Supplementary figures and images for: Validation of a simplified small-scale DNA extraction protocol from wine by quantitative real-time PCR
Source: 3 Biotech. 2024 May 2;14(5):145. doi: 10.1007/s13205-024-03992-x (PMC11065827; doi:10.1007/s13205-024-03992-x)

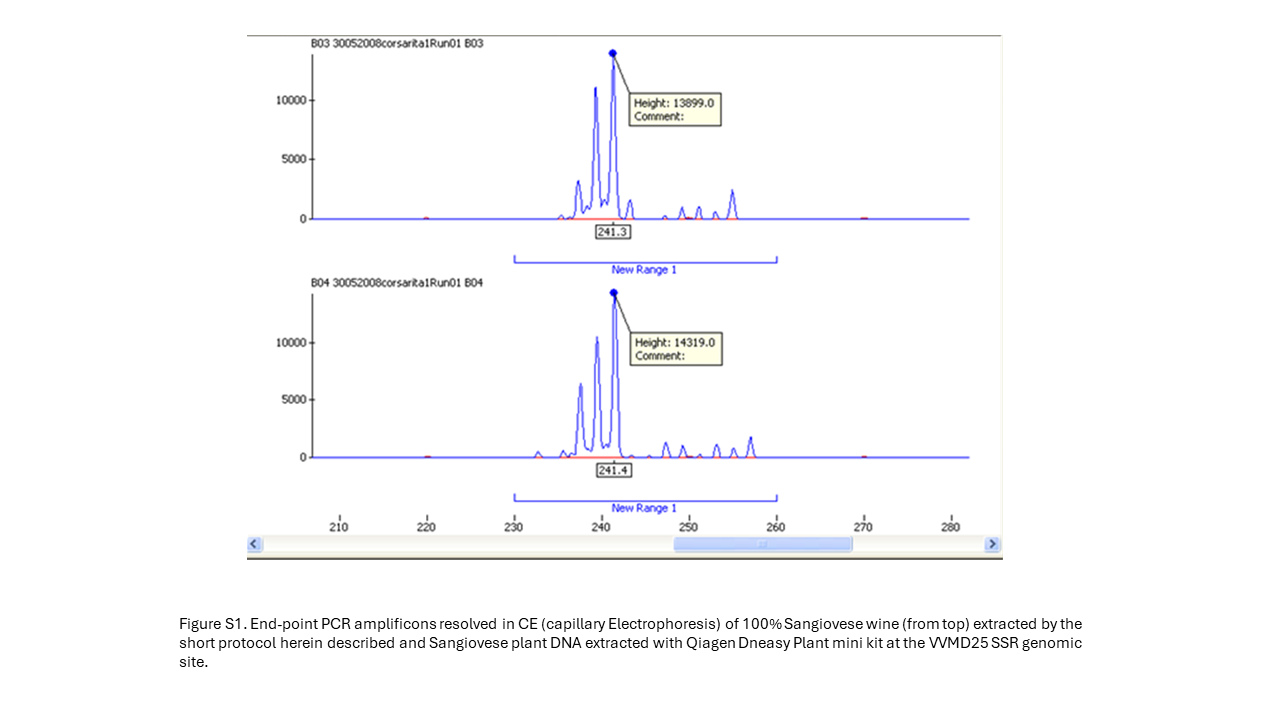

Supplement: Supplementary file 1 — Supplementary file1 (TIF 131 KB) [file 13205_2024_3992_MOESM1_ESM.tif]

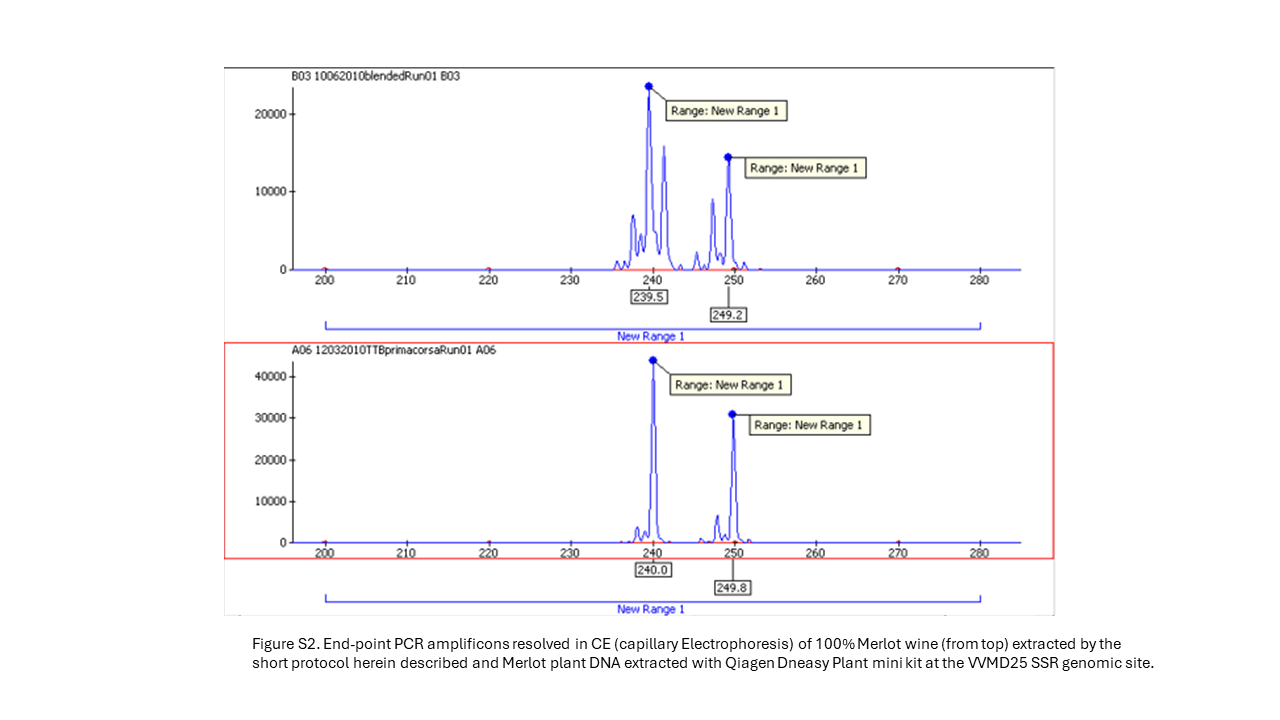

Supplement: Supplementary file 2 — Supplementary file2 (TIF 153 KB) [file 13205_2024_3992_MOESM2_ESM.tif]
